# Activities of Cefiderocol with Simulated Human Plasma Concentrations against Carbapenem-Resistant Gram-Negative Bacilli in an *In Vitro* Chemostat Model

**DOI:** 10.1128/AAC.01128-20

**Published:** 2020-10-20

**Authors:** Shuhei Matsumoto, Sachi Kanazawa, Takafumi Sato, Yoshinori Yamano

**Affiliations:** a Business Development, Shionogi & Co., Ltd., Osaka, Japan; b Drug Discovery & Disease Research Laboratory, Shionogi & Co., Ltd., Osaka, Japan

**Keywords:** cefiderocol, carbapenem resistant, *Enterobacterales*, *Acinetobacter baumannii*, chemostat model, nonfermenters

## Abstract

Activities of cefiderocol under simulated human plasma concentrations at the recommended dosing regimen of 2 g every 8 h with a 3-h infusion were evaluated using an *in vitro* chemostat model. Against a total of 6 meropenem-resistant Gram-negative strains with cefiderocol MICs of 0.5 to 4 μg/ml, including metallo-β-lactamase producers and carbapenem-resistant Acinetobacter baumannii, cefiderocol treatment showed a bactericidal effect within 8 h and sustained efficacy with no marked bacterial regrowth over 24 h.

## TEXT

Cefiderocol, a novel parenteral siderophore cephalosporin, shows potent activities against a wide range of multidrug-resistant Gram-negative bacilli (1–3). Especially, cefiderocol is the only β-lactam antibiotic with activities against both Gram-negative pathogens harboring metallo-type β-lactamase (MBL) and carbapenem-resistant Acinetobacter species (4). The United States Food and Drug Administration (FDA) approved cefiderocol for the treatment of complicated urinary tract infections with susceptible breakpoints of 2 and 1 μg/ml against *Enterobacterales* and Pseudomonas aeruginosa, respectively (5). The susceptibility breakpoint was different from the provisional breakpoint of 4 μg/ml against *Enterobacterales*, P. aeruginosa, Acinetobacter baumannii, and Stenotrophomonas maltophilia by the Clinical and Laboratory Standards Institute (6). In Europe, cefiderocol has been approved recently for the treatment of aerobic Gram-negative infections in adult patients with limited treatment options, and the European Committee on Antimicrobial Susceptibility Testing has set up breakpoints at 2 μg/ml for both *Enterobacterales* and P. aeruginosa. The purpose of this study is to evaluate the efficacy of cefiderocol under simulated human plasma concentrations over 24 h against clinical strains with a cefiderocol MIC around the breakpoint in a one-compartment *in vitro* chemostat model. MBL-producing strains were also included in this study because of the limited treatment options for such bacteria, and difficulties in evaluation by animal models have been reported due to the *in vitro-in vivo* discordance against MBL producers caused by the poor activity of MBL in animal models (7). Overall, this chemostat model is expected to provide useful information for treatment against isolates with cefiderocol MICs of 0.5 to 4 μg/ml, including MBL producers.

**FIG 1:**
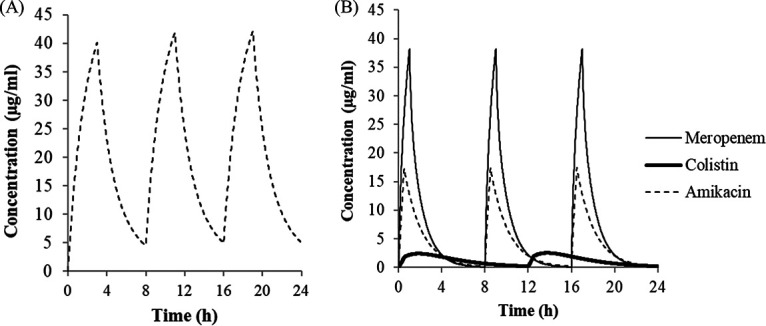
Concentration-time curves of cefiderocol (A) and colistin, amikacin, and meropenem (B) in a one-compartment *in vitro* chemostat model. For cefiderocol and meropenem, free concentrations corrected by plasma protein binding ratio were used.

A total of 6 clinical isolates (2 P. aeruginosa, 1 A. baumannii, 1 Escherichia coli, and 2 Klebsiella pneumoniae) harboring either VIM, IMP, OXA-23, NDM, or KPC, with cefiderocol MICs of 0.5 to 4 μg/ml using iron-depleted cation-adjusted Mueller-Hinton broth (ID-CAMHB) (Table 1) (8), were evaluated by the *in vitro* chemostat model as reported previously (9). The isolates of *Enterobacterales* and P. aeruginosa were selected to have the MIC that was around the susceptible breakpoint by FDA, and one isolate of A. baumannii was selected to have a similar MIC to the test isolates of P. aeruginosa. Briefly, an exponentially growing bacterial suspension of 5.20 × 10^5^ to 5.79 × 10^5^ CFU/ml was prepared and then incubated at 37°C for 24 h with or without the simulated human plasma concentrations of each antimicrobial agent (see Text S1 in the supplemental material). The plasma concentration-time curves were recreated as follows: 2 g cefiderocol every 8 h (i.e., three times a day [t.i.d.]) as a 3-h infusion, 2.5 mg/kg colistin methanesulfonate every 12 h as a 0.5-h infusion, 5 mg/kg amikacin t.i.d. as a 0.5-h infusion, and 1 g meropenem t.i.d. as a 1-h infusion (Fig. 1). For cefiderocol and meropenem, free concentrations corrected by plasma protein binding ratio were used. For colistin and amikacin, total concentrations were used because either total or free concentrations have been shown to be important for pharmacokinetics/pharmacodynamics (PK/PD) by several different reports (10–13). One of the colistin-based combination therapies was used as a positive control because this is an important currently available option for combatting carbapenem-resistant Gram-negative pathogens, including MBL producers (14, 15). As the isolates used in this study were resistant to multiple classes of antibiotics, colistin-amikacin combination therapy was evaluated, although these isolates were resistant to amikacin. Meropenem was used as a negative control because all test isolates were resistant to carbapenem.

**TABLE 1:** MICs of cefiderocol, colistin, amikacin, and meropenem against the test strains

Strain	Acquired β-lactamase(s)	MIC (μg/ml) of:				
		Cefiderocol	Colistin/amikacin^*a*^	Colistin	Amikacin	Meropenem
P. aeruginosa						
NUBL-7808	VIM-2	0.5	0.25	0.5	>32	>32
SR27001	IMP-1	1	0.5	2	>32	>32
A. baumannii SR08626	OXA-23	0.5	0.125	0.5	>32	32
E. coli DU48916	NDM-1, CTX-M-15, CMY-2, OXA-1	4	0.25	0.25	>32	32
K. pneumoniae						
SR08667	KPC-3, SHV	2	2	1	>32	>32
VA-384^*b*^	KPC-2, TEM-1, SHV-11, SHV-12	4	0.125	0.25	16	>32

a The MIC of colistin with 4 μg/ml amikacin was evaluated.

b Information on K. pneumoniae strains harboring β-lactamases is provided in reference 19.

**FIG 2:**
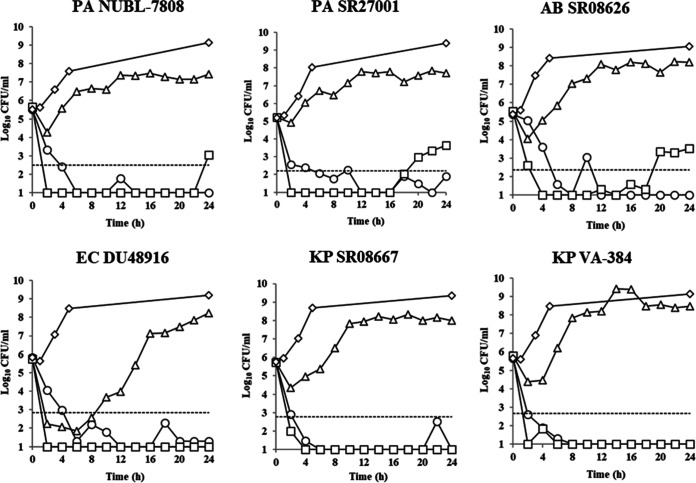
Number of viable cell-time curves exposed to the bacterial suspension under simulated human plasma concentrations of cefiderocol (open circles), colistin plus amikacin (squares), and meropenem (triangles) over 24 h. Each human dosing regimen was as follows: 2 g cefiderocol every 8 h as a 3-h infusion, 2.5 mg/kg colistin methanesulfonate every 12 h as a 0.5-h infusion, 5 mg/kg amikacin every 8 h as a 0.5-h infusion, and 1 g meropenem every 8 h as a 1-h infusion, respectively. Vehicle treatment is represented by diamonds. The 3-log_10_-CFU kill from the initial inoculum is represented by dotted horizontal lines. CFU, colony-forming units; PA, P. aeruginosa; AB, A. baumannii; EC, E. coli; KP, K. pneumoniae.

Against these carbapenem-resistant isolates, cefiderocol treatment showed potent activity that was comparable to that of colistin-based combination treatment (Fig. 2). Cefiderocol treatment showed more than a 3-log_10_ kill from the initial inoculum within 8 h posttreatment; thereafter, the 3-log_10_ kill was sustained until 24 h. The change of log_10_ CFU/ml from the initial inoculum after 24 h treatment was −3.30 to −4.77. The treatment by colistin plus amikacin caused more than a 3-log_10_ kill from the initial inoculum within 8 h posttreatment as well. However, the 3-log_10_ kill from the initial inoculum at 24 h posttreatment was not achieved in 3 nonfermenters as the number of the viable bacteria increased again after 20 h of treatment. The change of viable cells from the initial inoculum at 24 h posttreatment was −1.57 to −4.79. In contrast, meropenem treatment did not show the sustained bacterial killing during the treatment period and even the static effect after 24 h of treatment. The change of viable cells from the initial inoculum at 24 h posttreatment was 1.79 to 2.67.

This is the first report on the evaluation of cefiderocol efficacy under simulated human PK using an *in vitro* chemostat model. The results of the *in vitro* chemostat model using iron-depleted cation-adjusted Mueller-Hinton broth (ID-CAMHB) were consistent with the observation from several *in vivo* animal studies, which have been used to evaluate the potential use of cefiderocol to treat the infections caused by carbapenem-resistant Gram-negative bacteria with a MIC of ≤4 μg/ml. The PK/PD studies using mouse thigh infection models caused by a variety of Gram-negative bacteria showed that a 1-log_10_ reduction in bacteria burden at 24 h was associated with 75% of the percentage of time that free cefiderocol concentrations are above the MIC (%*fT*_MIC_) on average (16). The Monte-Carlo simulation showed that a dose of 2 g every 8 h as a 3-h infusion provided >90% probability of target attainment with a pharmacodynamic target of 75 or 100% %*fT*_MIC_ for a MIC of ≤4 μg/ml in nosocomial pneumonia patients (17). This potent efficacy was also confirmed by the efficacy studies using mouse thigh infection models under humanized PK (18). The data from this study further confirm efficacy against the isolates with a MIC of ≤4 μg/ml, which was consistent with these *in vivo* studies. It should be noted that this study provided the efficacy against NDM producers with a MIC of 4 μg/ml, which have never been appropriately evaluated due to the discrepancy between *in vitro* and *in vivo* studies. The good correlation of efficacy was observed between *in vitro* chemostat models using ID-CAMHB and *in vivo* studies, suggesting that these models could be used for further evaluation of efficacy and emergence of resistance during therapy against a variety of pathogens.

In summary, activities of the simulated human plasma concentrations of cefiderocol against strains with cefiderocol MICs of 0.5 to 4 μg/ml were evaluated in an *in vitro* chemostat model. The cefiderocol regimen showed bactericidal activities against carbapenemase-harboring carbapenem-resistant isolates of P. aeruginosa, A. baumannii, E. coli, and K. pneumoniae. These activities were comparable to that of a colistin-based regimen, suggesting cefiderocol as one of the therapeutic options for the treatment of infections caused by carbapenem-resistant Gram-negative pathogens, including MBL producers.

## Supplementary Material

Supplemental file 1
