# Supplementary material for: Activities of Cefiderocol with Simulated Human Plasma Concentrations against Carbapenem-Resistant Gram-Negative Bacilli in an In Vitro Chemostat Model
Source: Antimicrob Agents Chemother. 2020 Oct 20;64(11):e01128-20. doi: 10.1128/AAC.01128-20 (PMC7577172; doi:10.1128/AAC.01128-20)
Supplement: Supplemental file 1 [file AAC.01128-20-s0001.pdf]

Materials and Methods

Antimicrobial agents

Total of 4 antimicrobial agents were used in this study. Cefiderocol was synthesized by Shionogi & Co., Ltd. (Osaka, Japan). Colistin sulfate was obtained from Wako pure chemical industries, Ltd. (Osaka, Japan). Amikacin and meropenem were obtained from USP (Rockville, MD).

Test organisms

Six clinical isolates (2 *P. aeruginosa*, 1 *A. baumannii*, 1 *E. coli*, and 2 *K. pneumoniae*) were used in this study. All these isolates harbored at least 1 carbapenemase such as VIM, IMP, OXA-23, NDM, and KPC. MIC studies were conducted with three replicates, and the modal MICs were represented. For cefiderocol, iron-depleted (ID) cation-adjusted Mueller Hinton broth (CAMHB) with zinc concentration of 0.65 µg/ml was used as the test medium (1, 2). MICs of cefiderocol were 0.5 to 4 µg/ml. MICs of colistin, amikacin, and meropenem were determined by using broth microdilution method according to CLSI guidelines (2). MICs of colistin in combination with 4 µg/ml amikacin were 0.125 to 2 µg/ml. All test strains showed colistin-susceptible profile with colistin MIC of ≤ 2 µg/ml, however, those isolates were meropenem- and amikacin-resistant profiles. MICs of meropenem and amikacin were ≥ 16 µg/ml.

In vitro pharmacodynamic model

We used a one-compartment *in vitro* chemostat model which is the auto-simulation Shionogi dilution-type equipment (3) modified the model described by Grasso et al. (4).

The concentration-time curves were simulated by addition of the antimicrobial solution cooled at 4°C to the bacterial suspension or by dilution of the bacterial suspension with the test medium. To keep the volume of bacterial culture constant (50 ml), the culture solution was automatically removed under computer control. The bacterial suspension was prepared as follows. Overnight culture grown on tryptic soy agar plate was suspended in saline, and then measured the optical density at 625 nm (OD625) by spectrophotometer. In this study, the number of bacterial cells was assumed to be 1×10^8 colony-forming unit (CFU)/ml when OD625 was 0.1. The bacterial suspension was diluted with ID-CAMHB to prepare the bacterial suspensions of 5.20×10^5 to 5.79×10^5 CFU/ml.

The antimicrobial solution was prepared as follows. Cefiderocol was dissolved and diluted with saline to make 1000 µg/ml solution. Colistin and amikacin were dissolved with distilled water and further diluted with saline to make 1000 µg/ml and 350 µg/ml solutions, respectively. Meropenem was dissolved with 50 mM 3-(N-morpholino) propanesulfonic acid buffer (pH7.0) and further diluted with saline to make 760 µg/ml solution. In this study, the simulated human plasma concentrations in total 3 treatment regimen (cefiderocol, colistin in combination with amikacin, and meropenem) were recreated in the bacteria culture. Cefiderocol concentrations after intravenous administration of 2g cefiderocol as a 3-h infusion at 8-h intervals over 24 h were simulated based on the plasma concentrations after single intravenous administration of 2 g cefiderocol as a 1-h infusion to healthy volunteers (5), and then these concentrations were corrected by the reported plasma protein binding ratio in humans (58%) (6). Concentration curve of colistin was based on the 2.5 mg/kg colistin methanesulfonate

sodium single dose treatment administered as a 0.5-h infusion (7). The plasma concentrations-time curve of amikacin is based on the 5 mg/kg single dose treatment administered as a 0.5-h infusion (8). In the case of colistin, appropriate dosing regimens can be designed for validation in clinical setting with robust pharmacokinetics (PK) and pharmacodynamics (PD) data and associated PK/PD modeling by using the total drug concentration (9). The optimal initial dosing regimen of amikacin in clinical was set by PK/PD with total drug concentration (10). Meropenem concentrations after intravenous administration of 1g meropenem as a 1-h infusion at 8-h intervals over 24 h were simulated based on the plasma concentrations after single intravenous administration of 1 g meropenem to healthy volunteers (11), and then these concentrations were corrected by the reported plasma protein binding ratio in humans (12.8%) (12). The PK parameters to recreate the human plasma concentrations in the auto-simulation Shionogi dilution- type system were calculated by compartment model analysis. Each PK parameter is shown in Table S1.

Bacteria suspension was incubated at 37°C over 24 h under each simulated human plasma concentrations-time curve. A 0.5 ml of the bacterial culture was collected at 1, 3, 5, 24 h for vehicle treatment and 2 h intervals over 24 h for drug treatment, diluted with 4.5 ml of test medium supplemented with 1 mg/ml ferric ammonium citrate, and then stored at 4°C until determination of the viable cell counts. Collected samples were 10-fold serially diluted with saline or ID-CAMHB. A 0.01 or 0.1 to 0.5 ml of diluted bacterial cell suspensions were incubated in test medium or on agar plate (cation-adjusted Mueller Hinton agar plate for meropenem and colistin/amikacin treatment or ID-cation-adjusted Mueller Hinton agar for cefiderocol treatment) supplemented with 1

mg/ml ferric ammonium citrate, respectively, and then, the number of the colonies was counted. The effect of the carry-over of the antibiotics from the culture was considered to be negligible as the final concentration of the agar medium was below sub-MIC, which was confirmed by the results of serially diluted samples. When the number of viable cells per 1 ml of the bacterial suspension is 0, the value of $\log_{10}$ CFU/ml represents 1.

Table S1 Pharmacokinetic parameters of cefiderocol, colistin, amikacin, and meropenem

Drug	Human dosing regimen	Pharmacokinetic parameters			
		Vc (L)	α (h^{-1})	β (h^{-1})	kel (h^{-1})
Cefiderocol	2 g every 8-h as a 3-h infusion	9.846	0.763	0.268	0.518
Colistin*	2.5 mg/kg colistin methanesulfonate every 12-h as a 0.5-h infusion	1.694	0.602	0.313	0.097
Amikacin*	5 mg/kg every 8-h as a 0.5-h infusion	14.95	NA	NA	0.610
Meropenem	1 g every 8-h as a 1-h infusion	9.407	6.67	0.856	1.79

*: Colistin in combination with amikacin was evaluated in a one-compartment *in vitro* chemostat model.

Vc: distribution volume of central compartment, α: elimination rate constant in the distribution phase, β: elimination rate constant in the terminal phase, kel: elimination rate constant from central compartment, NA: not applicable.

Table S2 Change of viable cells from the initial inoculum after cefiderocol, colistin/amikacin, and meropenem treatment after 24 h treatment

Strain	Initial inoculum	Change of $\log_{10}$ CFU/ml from the initial inoculum after 24 h treatment		
		Cefiderocol	Colistin/Amikacin	Meropenem
P. aeruginosa				
NUBL-7808	5.59 ± 0.08	-4.51	-2.59	+1.79
SR27001	5.20 ± 0.00	-3.30	-1.57	+2.49
A. baumannii				
SR08626	5.45 ± 0.10	-4.38	-2.03	+2.65
E. coli				
DU48916	5.79 ± 0.05	-4.53	-4.73	+2.50
K. pneumoniae				
SR08667	5.76 ± 0.02	-4.77	-4.73	+2.27
VA-384	5.71 ± 0.07	-4.66	-4.79	+2.67

CFU: colony-forming units.

References

1. Clinical and Laboratory Standards Institute (CLSI), 2020. Performance Standards for Antimicrobial Susceptibility Testing; 30th Edition. CLSI Document M100-ED30. Clinical and Laboratory Standards Institute, Wayne, PA.
2. European Committee on Antimicrobial Susceptibility Testing (EUCAST), 2020. Breakpoints for cefiderocol from EUCAST. https://www.eucast.org/fileadmin/src/media/PDFs/EUCAST_files/Breakpoint_tables/Addenda/Cefiderocol_addendum_20200501.pdf.
3. Homma T, Hori T, Ohshiro M, Maki H, Yamano Y, Shimada J, Kuwahara S. 2010. In vitro pharmacokinetic and pharmacodynamic evaluation of S-013420 against Haemophilus influenzae and Streptococcus pneumoniae. Antimicrob Agents Chemother Oct;54(10):4300-5.
4. Grasso S, Meinardi G, de Carneri I, Tamassia V. 1978. New *in vitro* model to study the effect of antibiotic concentration and rate of elimination on antibacterial activity. Antimicrob Agents Chemother Apr;13(4):570-6.
5. Saisho Y, Katsube T, White S, Fukase H, Shimada J. 2018. Pharmacokinetics, Safety, and Tolerability of Cefiderocol, a Novel Siderophore Cephalosporin for Gram-Negative Bacteria, in Healthy Subjects. Antimicrobial Agents Chemother Feb 23;62(3):e02163-17.
6. Matsumoto S, Singley CM, Hoover J, Nakamura R, Echols R, Rittenhouse S, Tsuji M, Yamano Y. 2017. Efficacy of Cefiderocol against Carbapenem-Resistant Gram-Negative Bacilli in Immunocompetent-Rat Respiratory Tract Infection Models

Recreating Human Plasma Pharmacokinetics. Antimicrob Agents Chemother Aug 24;61(9):e00700-17.

7. Mizuyachi K, Hara K, Wakamatsu A, Nohda S, Hirama T. 2011. Safety and pharmacokinetic evaluation of intravenous colistin methanesulfonate sodium in Japanese healthy male subjects. Curr Med Res Opin Dec;27(12):2261-70.
8. Grenier B, Autret E, Marchand S, Thompson R. 1987. Kinetic parameters of amikacin in cystic fibrosis children. Infection Jul-Aug;15(4):295-9.
9. Plachouras D, Karvanen M, Friberg LE, Papadomichelakis E, Antoniadou A, Tsangaris I, Karaiskos I, Poulakou G, Kontopidou F, Armaganidis A, Cars O, Giamarellou H. 2009. Population Pharmacokinetic Analysis of Colistin Methanesulfonate and Colistin After Intravenous Administration in Critically Ill Patients With Infections Caused by Gram-Negative Bacteria. Antimicrob Agents Chemother Aug;53(8):3430-6.
10. Kato H, Hagihara M, Hirai J, Sakanashi D, Suematsu H, Nishiyama N, Koizumi Y, Yamagishi Y, Matsuura K, Mikamo H. 2017. Evaluation of Amikacin Pharmacokinetics and Pharmacodynamics for Optimal Initial Dosing Regimen. Drugs R D Mar;17(1):177-187.
11. Nakashima M, Uematsu T, Kanamaru M, Ueno K. 1992. Clinical phase 1 study of meropenem. Chemotherapy Apr;40(S-1):258-75. http://fa.chemotherapy.or.jp/journal/jjc/40/Supplement1/40_258.pdf.
12. Sumita Y, Nouda H, Tada E, Kohzuki T, Kato M, Okuda T, Fukusawa M. 1992. Pharmacokinetics of meropenem, a new carbapenem antibiotics, parenterally

administered to laboratory animals. Chemotherapy Apr; 40(S-1): 123-131. http://fa.chemotherapy.or.jp/journal/jjc/40/Supplement1/40_123.pdf
